# Circulatory Support with Venoarterial ECMO Unsuccessful in Aiding Endogenous Diltiazem Clearance after Overdose

**DOI:** 10.1155/2014/969578

**Published:** 2014-08-17

**Authors:** Erin N. Frazee, Sarah J. Lee, Ejaaz A. Kalimullah, Heather A. Personett, Darlene R. Nelson

**Affiliations:** ^1^Hospital Pharmacy Services, Mayo Clinic, 200 1st SW, Rochester, MN 55905, USA; ^2^Division of Pulmonary and Critical Care Medicine, Mayo Clinic, 200 1st SW, Rochester, MN 55905, USA; ^3^Department of Emergency Medicine and Division of Pulmonary and Critical Care Medicine, Loyola University Medical Center, 2160 S 1st Avenue, Maywood, IL 60153, USA

## Abstract

*Introduction.* In cardiovascular collapse from diltiazem poisoning, extracorporeal membrane oxygenation (ECMO) may offer circulatory support sufficient to preserve endogenous hepatic drug clearance. Little is known about patient outcomes and diltiazem toxicokinetics in this setting. *Case Report.* A 36-year-old woman with a history of myocardial bridging syndrome presented with chest pain for which she self-medicated with 2.4 g of sustained release diltiazem over the course of 8 hours. Hemodynamics and mentation were satisfactory on presentation, but precipitously deteriorated after ICU transfer. She was given fluids, calcium, vasopressors, glucagon, high-dose insulin, and lipid emulsion. Due to circulatory collapse and multiorgan failure including ischemic hepatopathy, she underwent transvenous pacing and emergent initiation of venoarterial ECMO. The peak diltiazem level was 13150 ng/mL (normal 100–200 ng/mL) and it remained elevated at 6340 ng/mL at hour 90. Unfortunately, the patient developed multiple complications which resulted in her death on ICU day 9. *Conclusion.* This case describes the unsuccessful use of ECMO for diltiazem intoxication. Although past reports suggest that support with ECMO may facilitate endogenous diltiazem clearance, it may be dependent on preserved hepatic function at the time of cannulation, a factor not present in this case.

## 1. Introduction

The American Association of Poison Control Centers reported more than 100,000 cardiovascular medication poisonings in 2011, of which calcium channel blocker (CCB) overdoses were involved in approximately 60% of the fatal events [1]. In both mono- and mixed-exposures of CCBs, diltiazem, a lipophilic, protein-bound, hepatically cleared, nondihydropyridine CCB, carried a significant risk of fatality [2].

Supportive care remains the cornerstone of diltiazem overdose management. Targeted therapies include calcium, high-dose insulin euglycemia, glucagon, lipid emulsion, and cardiac pacing [3]. Enhanced elimination by hemodialysis, hemoperfusion, albumin dialysis, and plasma exchange variably impacts clinical outcomes [3–6]. Because of the inconsistent results of extracorporeal drug removal, there is instead an interest in preservation of endogenous drug clearance mechanisms (i.e., hepatic metabolism) through extracorporeal membrane oxygenation (ECMO-) mediated circulatory support. Little is known about the impact of ECMO on patient outcomes and diltiazem toxicokinetics in this setting.

Herein, we present an unsuccessful case of ECMO use for diltiazem overdose and review the existing literature on circulatory support for nondihydropyridine CCB poisonings.

## 2. Case Report

A 36-year-old, 68 kg, woman with a history of myocardial bridging syndrome and an unroofing procedure two years prior to admission presented with a two-day history of chest pain for which she self-medicated with 2.4 grams of sustained release (SR) diltiazem over the course of 8 hours. Neither subjective nor objective evidence suggested coingestion. On arrival to the ED, she was mentating appropriately, with a blood pressure (BP) of 102/56 mm Hg and a pulse of 77 beats per minute (bpm) in sinus rhythm (Table 1). She became hypotensive (BP 88/40 mm Hg) and was administered intravenous (IV) fluids, 5 mg IV glucagon and 3 g of IV calcium gluconate. Hypotension persisted; therefore, a norepinephrine infusion was started. High-dose insulin (1 unit/kg/hr insulin) and a 1.5 mL/kg bolus of lipid emulsion (20% Intralipid) were subsequently given and continued in the ICU (insulin titrated to 10 units/kg/hr within 5 hours of ICU admission; Intralipid infused at 0.5 mL/kg/hr) [7]. At admission to the ICU, she was alert and oriented although increasingly dyspneic. Activated charcoal and gastric lavage were not used given the elapsed time between ingestion and presentation, the patient's nausea and concerns about the risk of aspiration, and her suspected ileus with high vasopressor requirements. Despite escalating norepinephrine doses to 2 mcg/kg/min, with the addition of 1 mcg/kg/min epinephrine, vasopressin at 0.1 units/min, calcium chloride boluses and infusion, and 75 mcg/kg/hr (5 mg/hr) of glucagon, BP remained 80's/30's with a pulse of 60 bpm. Initial ICU laboratory variables showed ionized calcium of 4.4 mg/dL, potassium of 3.4 mmol/L, lactate of 2.72 mmol/L, glucose of 127 mg/dL, and pH of 7.27. She was intubated due to hypoxia secondary to pulmonary edema. Eight hours after ICU admission, she developed frequent prolonged sinus pauses requiring transvenous pacing via a pulmonary artery catheter. She continued to worsen with profound metabolic acidosis requiring continuous venovenous hemofiltration. Charcoal hemoperfusion and albumin dialysis were unavailable for use in this case. Her circulatory collapse was unresponsive to vasopressors and thus two doses of methylene blue (2 mg/kg each) were attempted without a sustained effect. Therefore, she underwent emergent placement of right atrial (40 Fr Medtronic DLP malleable single stage venous cannula, http://www.medtronic.com/) and ascending aorta cannulae (Medtronic EOPA aortic 22 Fr cannula) for initiation of central veno-arterial (V-A) ECMO 13 hours from ICU admission (25 hours after reported ingestion; Table 1). The ECMO circuit was the Cardiohelp device with the HLS Module Advanced 7.0 Bioline heparin-coated portable cardiopulmonary support system (http://www.cardiohelp-us.com/en/home/). The ECMO circuit was primed with 600 cc of Plasmalyte and 1000 U heparin. She was supported for a total of 190 hours.

**Figure 1:**
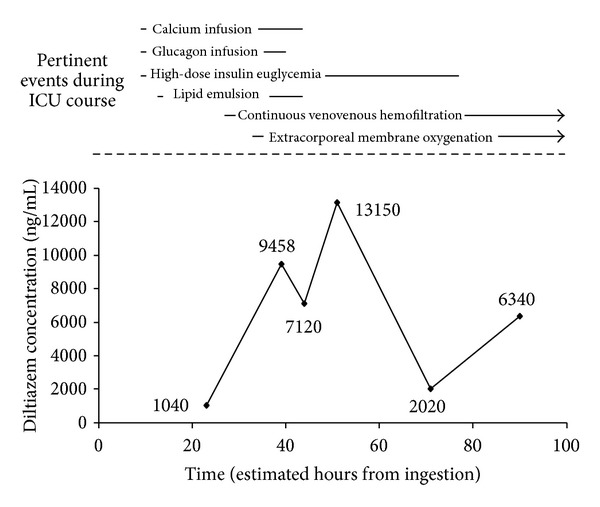
Diltiazem serum concentrations and concurrent interventions during ICU course according to suspected time from ingestion based on patient self-report.

Peak diltiazem serum concentration was 13150 ng/mL (Figure 1; therapeutic range 100–200 ng/mL; diltiazem concentrations determined with High Performance Liquid Chromatography with Ultraviolet Detection; MEDTOX Scientific, Inc., St. Paul, MN). Desacetyldiltiazem concentrations were not available. Seventy-one hours after ingestion, the diltiazem level decreased to 2020 ng/mL. To confirm downtrend, a diltiazem level 4 days after ingestion was obtained and found to have paradoxically increased to 6340 ng/mL.

Although she received full circulatory support with central V-A ECMO and high-dose vasopressors, she had an extremely low systemic vascular resistance and her course was complicated by global hypoperfusion including organ, limb, and tissue ischemia. She developed multifocal compartment syndrome, thrombocytopenia, uncontrolled gastrointestinal bleeding, and fulminant multiorgan failure leading to a transition to comfort care and death on ICU day 9.

## 3. Discussion

This report describes the course of a patient who experienced diltiazem SR poisoning and did not survive despite maximal medical therapy, transvenous pacing, CVVH, and venoarterial ECMO. She developed profoundly elevated diltiazem serum concentrations and an inconsistent drug decay curve after this overdose. The peak concentration noted was approximately 100-fold greater than the therapeutic range (100–200 ng/mL) and is at the upper limit of what has previously been reported [4–6, 8–11]. No consistent diltiazem elimination rate occurred and levels remained significantly elevated to hour 90 of admission. We hypothesize that the unusually prolonged drug exposure and timing of ECMO initiation relative to the development of hepatic dysfunction may have been critical factors in the patient's outcome.

Although the evidence is limited, circulatory support with ECMO for nondihydropyridine CCB poisonings could theoretically preserve end-organ function and facilitate endogenous hepatic clearance of the drug. Indeed, in 62 critically ill poisoned patients, 12 of whom ingested verapamil or diltiazem, individuals who underwent extracorporeal life support had a significantly better survival than those who did not (86% versus 48%, respectively; *P* = 0.02) [12]. Three published reports describe the use of ECMO for sustained release nondihydropyridine CCB poisoning in pediatric/neonatal patients. In two patients, CCB poisoning was associated with verapamil in combination with the nonselective beta-blocker propranolol. ECMO cannulation occurred at 4 and 10 hours after intoxication and both patients survived to hospital discharge [13, 14]. The third pediatric case reported is of a 16-year-old female with an acute intentional ingestion of 12 grams of sustained release diltiazem. She received gastric lavage, activated charcoal, and pacing. ECMO began 17 hours after the ingestion and at hour 22 she had evidence of mild hepatic dysfunction with peak AST and ALT of 320 and 197, respectively. After 48 hours of circulatory support, ECMO was terminated due to uncontrolled mediastinal hemorrhage, but the patient was able to survive to discharge [10]. Extracorporeal circulatory support has also infrequently been described in the setting of adult nondihydropyridine CCB intoxications [15–18]. A 41-year-old adult male intentionally ingested multiple medications including verapamil and was successfully managed with 5 hours of percutaneous cardiopulmonary bypass introduced 8 hours after the toxic ingestion [17]. Another case of a polyingestion which included 7.2 g of slow-release verapamil was initiated on ECMO in the emergency department and successfully managed with 6 days of support [15]. Lastly, over ten years at a single center, 17 patients with drug poisoning and shock were placed on extracorporeal life support. Four of these individuals ingested verapamil and received circulatory support within 5 hours of the ingestion. Ten (58%) patients in the overall group developed cannulation-related injuries of the femoral vessels and 13 (76%) patients survived. Two (50%) of the 4 patients with verapamil ingestions survived [16].

Whereas several previous reports have signaled a possible benefit on outcomes with ECMO use for nondihydropyridine CCB poisonings, the patient in this case died despite maximal medical therapy, CVVH to control refractory acidosis and hyperkalemia, and circulatory support with ECMO. Multiple factors may have contributed to this negative clinical outcome. Her course was complicated by delayed mesenteric ischemia which, in the setting of poisonings, has been shown to affect younger patients with fewer risk factors [19]. The diltiazem concentrations seen in this patient were markedly elevated and erratic which suggests that the patient was exposed to sustained toxic levels for a longer period of time than in previous reports. Prior kinetic analyses of SR diltiazem overdoses have demonstrated an elimination half-life of 13–48 hours [5, 10, 11]. Zero order kinetics appear to predominate at concentrations >650 ng/mL with a return to first order elimination below this threshold [6]. Durward and colleagues characterized serial diltiazem levels after overdose in a female patient who received 48 hours of ECMO. The admission diltiazem concentration was approximately 6000 ng/mL. Two rounds of charcoal hemoperfusion were attempted which resulted in temporary reductions in serum diltiazem concentrations with near complete rebound in the 8 hours after each run. By hour 120 after ingestion, her diltiazem concentration was <1000 ng/mL and she survived to be discharged from the hospital. In our case, stability of diltiazem elimination was not achieved and the serum concentration spontaneously rebounded from 2020 ng/mL at hour 71 after ingestion to 6340 ng/mL at hour 90.

The atypical concentration pattern and prolonged exposure we found may have been affected by a number of factors. Diltiazem could have accumulated in the gastrointestinal tract because the patient did not receive gastric decontamination or because of the development of decreased mesenteric perfusion or an ileus. The bowel intervention for mesenteric ischemia may have contributed to the 90-hour rebound in serum concentrations (Table 1). We also cannot exclude the possibility that the 2020 ng/mL serum concentration drawn at 71 hours after ingestion was spurious. The timing of lipid exposure makes it unlikely that an altered volume of distribution associated with this therapy impacted drug levels, but alterations in protein binding may have resulted in heightened serum concentrations of free drug. CVVH has not been shown to significantly contribute to diltiazem elimination and thus it is unlikely that this concurrent intervention altered the serum levels [20].

In addition to the potential for altered drug absorption and distribution, we also hypothesize that the patient may have experienced altered diltiazem metabolism. It is of particular importance to preserve hepatic perfusion in the case of diltiazem poisonings because the drug undergoes extensive hepatic metabolism via deacetylation and only 1–3% of drug is eliminated unchanged in the urine [20]. Previous reports have either not described liver function at the time of ECMO initiation or demonstrated only mild increases in transaminases. In this case, ECMO began 17 hours from admission (25 hours from ingestion), but there was already significant AST and INR elevation in the absence of therapeutic anticoagulation, suggesting acute ischemic hepatopathy. Mechanical circulatory support to preserve hemodynamics and consequently endogenous drug clearance may have been insufficient to overcome this preexisting organ dysfunction. It is possible that introduction of ECMO prior to the onset of hepatic dysfunction may have resulted in improved perfusion and consequent drug clearance.

This case report of a SR diltiazem overdose describes drug toxicokinetics and the role for ECMO as a therapeutic intervention. We documented prolonged exposure to toxic diltiazem levels and an inconsistent drug decay curve, likely attributable to altered absorption, distribution, and metabolism. Although ECMO may theoretically facilitate endogenous diltiazem clearance, the timing of initiation may be a key determinant in its success. This patient exhibited signs of hepatic failure before ECMO initiation. This may have decreased the likelihood that enhanced circulatory support could facilitate sufficient endogenous drug clearance to offset the risks of the intervention. Future study is indicated to determine if early ECMO initiation in SR diltiazem overdoses improves patient outcomes.

## Figures and Tables

**Table 1:** Vital signs and laboratory measures and events during the admission.

Hours and days after ingestion	Vitals signs and pertinent laboratory values	Events and interventions	Diltiazem level (ng/mL)
8 hours (presentation)	Vitals: HR 77 bpm, BP 102/56 mmHg (MAP 71)	Calcium, glucagon, high-dose insulin, fluids, lipid emulsion, and vasopressors started	—

19 hours	Vitals: HR 78 bpm, supported BP 112/38 mmHg (MAP 53) PAC: CI 5.7 L/min/m^2^, SVRi 535 dynes*·*sec/cm^5^/m^2^ ABG: pH 6.96, pCO_2_ 41 mmHg, pO_2_ 110, HCO_3_ 9 mmol/L Labs: lactate 8.7 mmol/L; potassium 3.1 mmol/L; glucose 430 mg/dL; AST 79 U/L	PAC placed, transvenously paced at 80 bpm due to interval development of prolonged sinus pauses; methylene blue attempted; CVVH begun	—

23 hours	—	—	1140

25 hours	Immediately before ECMO cannulation: Vitals: HR 96 bpm (paced), supported BP 93/48 mmHg (MAP 63) PAC: CI 4.1 L/min/m^2^, SVRi 804 dynes*·*sec/cm^5^/m^2^ Labs: lactate >12.2 mmol/L; potassium 2.2 mmol/L; AST 3112 U/L, INR 2.4	V-A ECMO cannulation, total circuit flow 4.8–5.1 L/min (ECMO CI 2.9–3.1 L/min/m^2^)	—

39 hours	—	—	9450

44 hours	—	—	7120

51 hours	Hemodynamics: HR 100 (paced; asystolic when pacemaker is off), total ECMO circuit flow 4.6 L/min (ECMO CI 2.7 L/min/m^2^) Labs: lactate >18 mmol/L; potassium 4.9 mmol/L	Abdominal compartment syndrome, to operating room for exploration, evacuation of ascites, and temporary closure	13150

71 hours	Labs: lactate 15.4 mmol/L; potassium 8.1 mmol/L; aPTT 63 seconds (heparinized), INR 2.3 TTE: LV EF 10–15% and a severe decrease in right ventricular systolic function; no evidence of tamponade	Persistent elevation in potassium and lactate with increasing abdominal distension; prompted exploration where ischemic small bowel and colon were found along with a large retroperitoneal hematoma; resected and left in discontinuity	2020

90 hours	—	Developed bilateral lower-extremity compartment syndrome requiring fasciotomies	6340

Day 5	—	Underwent abdominal reexploration with creation of an end ileostomy and 3 mucous fistulae	—

Day 7	—	Regained sinus rhythm and downtitrated vasopressors; unable to wean from ECMO	—

Day 8	—	Developed a GI bleed in the setting of refractory thrombocytopenia, anticoagulation for ECMO, and autoanticoagulation from acute liver injury	—

Day 9	—	Transitioned to comfort cares and died	—

HR: heart rate; BP: blood pressure, MAP: mean arterial pressure; PAC: pulmonary artery catheter; CI: cardiac index; SVRi: systemic vascular resistance index; ABG: arterial blood gas; CVVH: continuous venovenous hemofiltration; V-A ECMO: venoarterial extracorporeal membrane oxygenation; AST: aspartate aminotransferase; INR: international normalized ratio; LV EF: left ventricular ejection fraction.
